# 1-h Glucose During Oral Glucose Tolerance Test Predicts Hyperglycemia Relapse-Free Survival in Obese Black Patients With Hyperglycemic Crises

**DOI:** 10.3389/fendo.2022.871965

**Published:** 2022-06-02

**Authors:** Ram Jagannathan, Darko Stefanovski, Dawn D. Smiley, Omolade Oladejo, Lucia F. Cotten, Guillermo Umpierrez, Priyathama Vellanki

**Affiliations:** ^1^ Division of Hospital Medicine, Emory University School of Medicine, Atlanta GA, United States; ^2^ Department of Biostatistics, University of Pennsylvania School of Veterinary Medicine, Kennett Square, PA, United States; ^3^ Division of Endocrinology, Metabolism and Lipids, Emory University School of Medicine, Atlanta, GA, United States

**Keywords:** diabetic ketoacidosis, 1-h and 2-h glucose values, stress hyperglycemia, oral glucose tolerance, net reclassification improvement, ROC (receiver operating characteristic curve)

## Abstract

**Objective:**

Approximately 50% of obese Black patients with unprovoked diabetic ketoacidosis (DKA) or severe hyperglycemia (SH) at new-onset diabetes achieve near-normoglycemia remission with intensive insulin treatment. Despite the initial near-normoglycemia remission, most DKA/SH individuals develop hyperglycemia relapse after insulin discontinuation. Traditional biomarkers such as normal glucose tolerance at the time of remission were not predictive of hyperglycemia relapse. We tested whether 1-h plasma glucose (1-h PG) at remission predicts hyperglycemia relapse in Black patients with DKA/SH.

**Methods:**

Secondary analysis was performed of two prospective randomized controlled trials in 73 patients with DKA/SH at the safety net hospital with a median follow-up of 408 days. Patients with DKA/SH underwent a 5-point, 2-h 75-g oral glucose tolerance test after hyperglycemia remission. Hyperglycemia relapse is defined by fasting blood glucose (FBG) > 130 mg/dl, random blood glucose (BG) >180 mg/dl, or HbA1c > 7%.

**Results:**

During the median 408 (interquartile range: 110–602) days of follow-up, hyperglycemia relapse occurred in 28 (38.4%) participants. One-hour PG value ≥199 mg/dl discriminates hyperglycemia relapse (sensitivity: 64%; specificity: 71%). Elevated levels of 1-h PG (≥199 mg/dl) were independently associated with hyperglycemia relapse (adjusted hazard ratio: 2.40 [95% CI: 1.04, 5.56]). In a multivariable model with FBG, adding 1-h PG level enhanced the prediction of hyperglycemia relapse, with significant improvements in C-index (Δ: +0.05; p = 0.04), net reclassification improvement (NRI: 48.7%; p = 0.04), and integrated discrimination improvement (IDI: 7.8%; p = 0.02) as compared with the addition of 2-h PG (NRI: 20.2%; p = 0.42; IDI: 1.32%; p = 0.41) or HbA1c (NRI: 35.2%; p = 0.143; IDI: 5.8%; p = 0.04).

**Conclusion:**

One-hour PG at the time of remission is a better predictor of hyperglycemia relapse than traditional glycemic markers among obese Black patients presenting with DKA/SH. Testing 1-h PG at insulin discontinuation identifies individuals at high risk of developing hyperglycemia relapse.

## Introduction

Approximately 50% of obese Black patients with new-onset, unprovoked diabetic ketoacidosis (DKA) or severe hyperglycemia (SH) achieve near-normoglycemia remission (defined as fasting blood glucose [FBG] <130 mg/dl, random blood glucose (BG) <180 mg/dl, and HbA1c < 7% while off insulin for at least 1 week) with aggressive insulin treatment (1). These patients exhibit clinical, metabolic, genetic, and autoimmune features consistent with type 2 diabetes. Although exact pathophysiologic mechanisms are unknown, near-normoglycemia remission is achieved in this patient population due to improved pancreatic beta (β)-cell function and insulin sensitivity (2–4). Glycemic control after near-normoglycemia remission is variable. Over the long-term, most patients experience pancreatic β-cell function failure resulting in the need for antidiabetic medications (4–6), while less than 10% of patients are able to maintain remission without medication over ~8 years (3). Despite the initiation of antidiabetic medications, 73% experience hyperglycemia relapse, and even DKA (4). Therefore, predictors of glycemic failure at the time of near-normoglycemia remission are needed to see which patients should have more aggressive treatment. We and others have shown that glycemic control consistent with glucose levels at near-normoglycemia remission can be maintained with monotherapy up to a median of 480 days (2, 6).

At near-normoglycemia remission, the clinical presentation and oral glucose tolerance test (OGTT) are heterogeneous, with 12%–17% having normal glucose tolerance (3, 7). However, normal glucose tolerance did not predict time in glycemic control (3, 7). Accumulating longitudinal evidence from epidemiological studies shows that a 1-h plasma glucose load (1-h PG) level >155 mg/dl during OGTT is a better predictor of type 2 diabetes and cardiovascular disease mortality than fasting glucose 2-h PG load or HbA1c (8–10). In addition, an elevated 1-h PG level is associated with decreased insulin secretion and sensitivity (11), impaired hepatic enzymes (12), and increased accentuation of reactive oxygen species generation (13, 14). However, the association of 1-h PG with hyperglycemia relapse in patients with DKA and SH at new-onset diabetes was never studied. In this study, we evaluated the association of the 1-h PG with the incident hyperglycemia relapse in obese Black patients presenting DKA and SH at new-onset diabetes over a mean follow-up period of 3 years. We also evaluated whether adding 1-h PG significantly improves the prediction of hyperglycemia relapse compared to glucose levels at other time points during the OGTT and HbA1c levels at the time of insulin discontinuation.

## Materials and Methods

### Participants

This study combined participants from two randomized controlled studies (NCT01099618 and NCT00426413) conducted between 2007 and 2014. The study design inclusion/exclusion criteria are detailed elsewhere (6, 7). Briefly, for both studies, participants with no prior history of diabetes presenting with DKA as defined by the American Diabetes Association (ADA) and SH (blood glucose >400 mg/dl without ketoacidosis) have consented during hospital admission. All subjects had glutamic decarboxylase-65 antibody measured to exclude autoimmune diabetes.

### Study Protocol

The Institutional Review Board at Emory University approved the combined analysis for both studies. After acute resolution of DKA or SH, all participants were treated intensively with subcutaneous insulin to a target fasting and pre-meal BG between 70 and 130 mg/dl (3.9–7.2 mmol/L). Insulin was titrated to achieve near-normoglycemia remission defined as FBG < 130 mg/dl (7.2 mmol/L) and random BG <180 mg/dl (10 mmol/L) and HbA1c < 7% (53 mmol/L) while off insulin therapy for at least 1 week. All participants then received a 75-g 120-min OGTT. After the OGTT, in one study (NCT01099618), participants were randomized into three groups: sitagliptin 100 mg daily (n = 16), metformin 1,000 mg daily (n = 17), or placebo (n = 15) (6). In the second study (NCT00426413), participants were randomized into two groups, pioglitazone 30 mg daily (n = 22) or placebo (n = 22), and followed up till hyperglycemia relapse (defined as FBG > 130 mg/dl (7.2 mmol/L), random BG >180 mg/dl (10 mmol/L) for a period of two consecutive days, or HbA1c ≥ 7% (53 mmol/L)). All participants were followed up until hyperglycemia relapsed or till the end of the study duration (~3 years) (2, 7).

### Study Measurements

Patient demographics and clinical characteristics were obtained from the electronic medical record and medical history during study visits. OGTTs were performed after at least an 8-h overnight fast. After fasting insulin and glucose levels were measured, 75 g of anhydrous glucose was ingested within 1 min. Glucose and insulin levels were then measured at 15, 30, 60, 90, and 120 min. Analyses of post-load glucose levels were focused on measurements at 1 h.

### Outcomes and Calculations

Hyperglycemia relapse was defined as FBG > 130 mg/dl, random BG >180 mg/dl on at least 2 consecutive days, or HbA1c > 7%. Glucose and insulin levels were used to calculate insulin sensitivity and secretion. Insulin sensitivity (Si) was calculated using oral minimal model analysis (6). Insulin secretion was calculated as the incremental area under the curve (AUCi) from insulin levels during the OGTT (6). The disposition index was calculated as the product of Si and AUCi.

### Statistical Analysis

Baseline characteristics of participants were expressed as means with SD or medians with interquartile ranges (IQRs) for continuous variables and numbers (proportions) with percentages for categorical variables. Since previous studies did not find a significant difference in insulin secretion and sensitivity after insulin discontinuation (6, 7), the data for this study were combined. Because this *post-hoc* analysis’s primary objective was to assess the predictive power of 1-h PG on incident hyperglycemia relapse, both control (placebo) and intervention groups (metformin, sitagliptin, or pioglitazone) were examined as a single cohort. For the time-to-event analysis, the follow-up length was calculated as the time from near-normoglycemia remission to the date of the first occurrence of hyperglycemia relapse or the last follow-up with the last censoring date of February 2014. To evaluate the optimal threshold of 1-h PG levels to predict hyperglycemia relapse, the 1-h PG levels were identified for the maximum of Youden’s Index, a summary statistic of the receiver operating characteristic (ROC) curve defined as (sensitivity + specificity − 1) (15). To minimize overfitting and to quantify optimism, specificity and sensitivity of the thresholds given were computed with 1,000 stratified bootstrap replicates with a 95% CI. The cutoff value of 1-h PG was appraised according to the least distance from the upper-left corner of the ROC curve. The Kaplan–Meier curves were generated to estimate the cumulative incidence of hyperglycemia relapse by the identified 1-h PG categories at the time of insulin discontinuation; a log-rank test was computed to compare survival distributions. For each subject, Cox proportional hazards models were used to estimate hazard ratios (HRs) and corresponding 95% CIs for incident hyperglycemia relapse associated with the baseline levels of 1-h PG levels. Proportionality hazard assumption in Cox models for all predictors and covariates in a fully adjusted multivariable model was assessed using the Schoenfeld residuals regressed against follow-up time; no violation of proportionality was observed. The biologically relevant or statistically significant variables in univariate analysis for the multivariable-adjusted models were chosen.

The incremental benefit of 1-h PG, 2-h PG, or HbA1c above and beyond the traditional risk factors (age, sex, body mass index (BMI), treatment allocation, and FBG) for predicting the risk of hyperglycemia relapse in patients with DKA/SH were assessed using a model fit, calibration, discrimination, and reclassification. Model fit was determined using the deviance analysis, with lower deviance, which means better model fit. Model calibration was determined using the Hosmer–Lemeshow goodness-of-fit test, with larger p-values (>0.05) indicating good agreement between observed and predicted outcomes. The AUC of the ROC was used to compute model discrimination. Improvement in AUC after adding the 1-h PG, 2-h PG, or HbA1c was estimated using the method of DeLong et al. (16, 17) Finally, continuous/category-free net reclassification improvement (NRI > 0) and absolute integrated discrimination improvement (IDI) was assessed to ascertain the enhanced predictability of glucose biomarkers on the hyperglycemia relapse outcomes (18).

A two-sided p-value of less than 0.05 was considered significant. Statistical analyses were performed using the survminer (version 0.4.7) (19), survival (version 3.2-3) (20), optimal cut points, PredictABEL (version 0.1), and tableone (version 0.10.0) packages in R (version 3.3.1).

## Results

### Cohort Description

Seventy-three participants with DKA (n = 40) and SH (n = 33) with near-normoglycemia remission who had OGTTs performed after insulin discontinuation were included in the analysis. The mean age was 46.9 ± 10.3 years, 26 (35.6%) were women, and the mean BMI was 36.1 ± 9.5 kg/m^2^. Based on the fasting and 2-h PG levels, 9 (12.1%) had normal glucose tolerance, 34 (45.9%) had prediabetes, and 30 (41.2%) had diabetes as defined by the ADA guidelines (21).

#### Association of 1-h Plasma Glucose Levels With Incident Hyperglycemic Relapse

During the median 408 (IQR: 110–602) days of follow-up, hyperglycemia relapse occurred in 28 (38.4%) participants. The cumulative incidence of hyperglycemia relapse was lower in those who received oral antidiabetic agents [pioglitazone, metformin, or sitagliptin; 14 (28.0%)] than in controls [placebo; n = 14 (60.9%)]. There was no significant difference in age, BMI, and the proportion of smokers and family history of diabetes between those who did and did not have hyperglycemia relapse. Participants who progressed to hyperglycemia relapse outcome had higher baseline values of FBG and 15-min, 1-h, and 2-h PG. In the crude model, the unadjusted HR per 1 SD change in plasma 1-h PG was 1.88 [95% CI: 1.25, 2.82] (**Table 1**) for hyperglycemia relapse. In the fully adjusted model including age, sex, randomization group (placebo vs. treated), BMI, and baseline diagnosis (DKA vs. SH), the independent association between 1-h PG and incident hyperglycemia relapse remained significant [adjusted HR (aHR): 1.98 (95% CI: 1.27, 3.09)].

**Table 1:** Hazard ratio for 1-h PG levels and hyperglycemia relapse.

Model	1-h PG as a continuous variable (per SD change)	1-h PG (≥199 vs. <199 mg/dl)
1	1.88 [1.25, 2.82]	2.50 [1.15, 5.43]
2	1.97 [1.27, 3.05]	2.43 [1.05, 5.62]
3	2.02 [1.29, 3.15]	2.42 [1.04, 5.61]
4	1.98 [1.27, 3.09]	2.40 [1.04, 5.56]

Data are expressed as hazard ratio (HR) (95% CI). The given HR is for 1 SD change in 1-h PG or 1-h PG categories (≥199 vs. <199 mg/dl) and continuous covariates in multivariable Cox regression analysis. Model 1, crude model; model 2, adjusted for age, sex, and intervention group; model 3, further adjusted for BMI; and model 4, further adjusted for baseline diagnosis (severe hyperglycemia vs. diabetic ketoacidosis).

PG, plasma glucose; BMI, body mass index.

#### 1-h Plasma Glucose Optimal Cut Point to Predict Hyperglycemia Relapse

Based on Youden’s analysis, a 1-h PG cutoff of 11.0 mmol/L (199 mg/dl) was found to differentiate the individuals with/without the development of hyperglycemia relapse. The sensitivity and specificity of the optimal cutoff value [11.0 mmol/L (199 mg/dl)] were 64% and 71%, respectively. Then, we dichotomize the 1-h PG values and estimated the association of 1-h PG categories [1-h PG_High_ ≥11.0 mmol/L [≥199 mg/dl); 1-h PG_Normal_ <11.0 mmol/L (<199 mg/dl)] on the risk of developing hyperglycemia relapse (**Table 1**). The aHR shows a 2.5-fold incidence of hyperglycemia relapse with a 1-h PG_High_ compared to 1-h PG_Normal_. Characteristics of participants according to the 1-h PG cut point are shown in **Table 2**. Participants who were in the 1-h PG_High_ category were significantly older and had higher FBG and 15-min, 30-min, and 2-h PG levels. Furthermore, the levels of Si were 76.8% (1.42 vs. 0.33), and DI was 70.0% (0.63 vs. 0.19) lower in individuals with 1-h PG_High_ than in those with 1-h PG_Normal_. We did not observe any significant differences in other variables between the 1-h PG_High_ and 1-h PG_Normal_ groups.

**Table 2:** Baseline characteristics of the study population, stratified according to 1-h plasma glucose challenge 199 mg/dl.

	1-h plasma glucose challenge	
	Normal (<199 m/dl, 11.0 mmol/L) n = 42	High (≥199 mg/dl, 11.0 mmol/L) n = 31
Age, years	44.43 (11.44)	50.23 (7.59)
Gender, male, n (%)	25 (59.5)	22 (71.0)
BMI, kg/m^2^	36.81 (10.65)	35.06 (7.62)
Family history of diabetes, n (%)	35 (83.3)	26 (83.9)
Baseline diagnosis, n (%)		
Severe hyperglycemia	22 (52.4)	18 (58.1)
Diabetic ketoacidosis	20 (47.6)	13 (41.9)
Treatment with an oral antidiabetic agent, n (%)		
No (placebo)	11 (26.2)	12 (38.7)
Yes (sitagliptin, pioglitazone, or metformin)	31 (73.8)	19 (61.3)
Glucose, mg/dl (mmol/L)		
Fasting	98 ± 15.41 (5.4 ± 0.8)	117 ± 18 (6.5 ± 1.0)
15 min	110 ± 20 (6.1 ± 1.1)	131 ± 33 (7.3 ± 1.8)
30 min	135 ± 29 (7.5 ± 1.6)	174 ± 41 (9.7 ± 2.3)
120 min	163 ± 42 (9.0 ± 2.3)	222 ± 55 (12.3 ± 3.1)
		
AUCi	5416.50 [2538.30, 7845.19]	4384.50 [2868.00, 6132.38]
Si	1.42 [0.56, 3.49]	0.33 [0.00, 0.84]
Di	0.63 [0.36, 1.43]	0.19 [0.00, 0.38]
Hyperglycemia relapse during follow-up, n (%)	10 (23.8)	18 (58.1)
The median time to hyperglycemia relapse, days (IQR)	427.50 [102.75, 598.75]	336.00 [116.00, 580.00]

Continuous variables are shown as mean ± SD or medians (IQR). Data for the categorical variables (gender, active smoking, family history of diabetes, and baseline diagnosis) are presented as counts and corresponding percentages.

IQR, interquartile range; BMI, body mass index; AUCi, area under the curve of insulin; Si, insulin sensitivity from the minimal model; and Di, disposition index.

#### Hazard Risk

**Figure 1:**
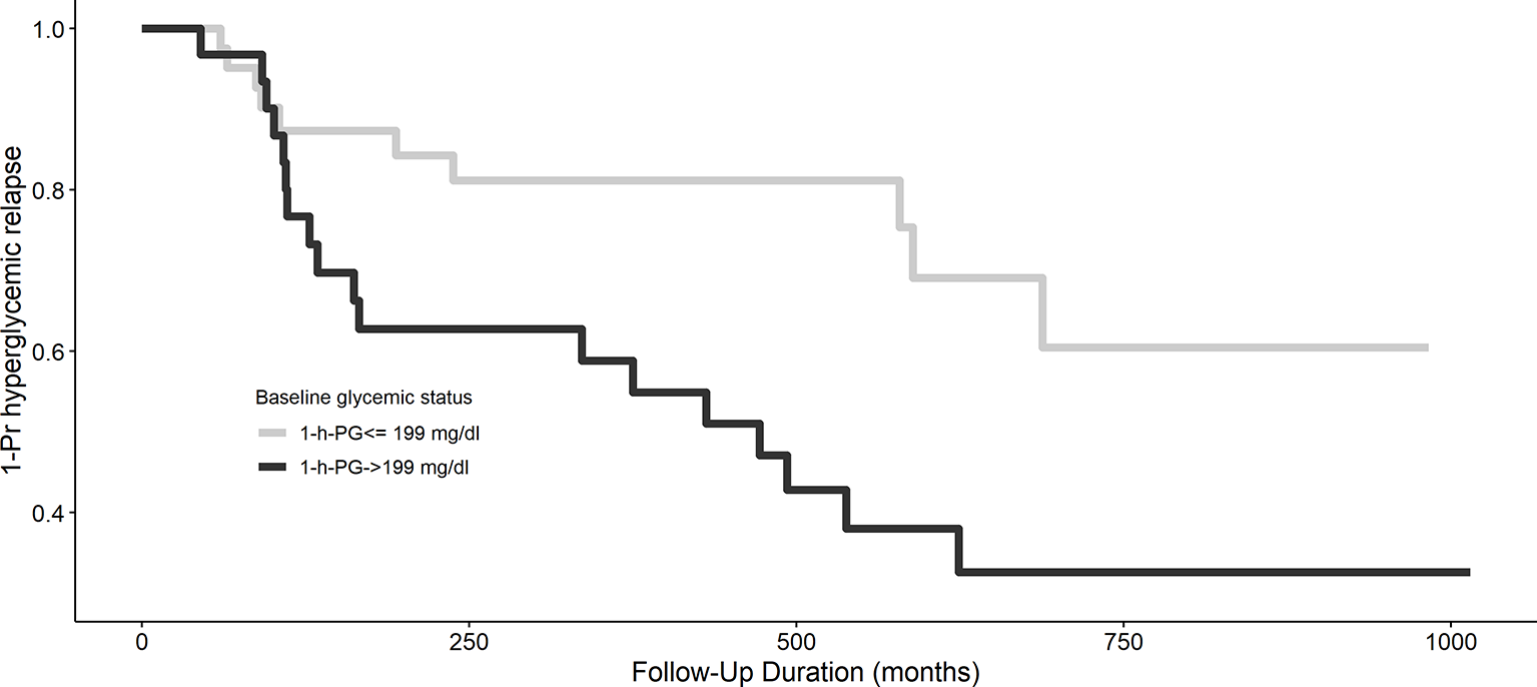
Kaplan–Meier curve of hyperglycemia relapse based on 1-h plasma glucose challenge categories. A 1-h plasma glucose challenge level ≥199 mg/dl is associated with longer hyperglycemia relapse-free survival, p = 0.02.

The Kaplan–Meier plot shows the unadjusted hyperglycemia relapse-free survival stratified based on 1-h PG levels (**Figure 1**). Individuals with 1-h PG_Normal_ (<199 mg/dl) had ~3 months of delayed median time to onset of hyperglycemia relapse than those with 1-h PG_High_ (≥199 mg/dl; log-rank test p = 0.02). Congruent with the above, both crude [HR: 2.50 (95% CI: 1.15,5.43)] and aHRs for the development of hyperglycemia relapse were significantly greater in the 1-h PG_High_ group [aHR: 2.40 (95% CI: 1.04, 5.56)] versus the 1-h PG_Normal_ (<199 mg/dl) group.

### Discriminative Ability

The results of five multivariate prognostic models [traditional (model 1) and traditional+1-h PG (model 2), traditional+2-h PG (model 3), traditional+HbA1c (model 4), and traditional+1-h PG+2-h PG (model 5)] are shown in **Table 3**. The addition of the 1-h PG to the traditional model containing age, sex, BMI, gender, treatment group, baseline diagnosis, and FBG improved model fit (Δ deviance: −5.60, p = 0.018), calibration (Hosmer–Lemeshow test, p > 0.05; **Supplementary Figure 1**), discrimination (AUC increase from 0.84 to 0.89, ΔC: +0.05; p = 0.039; **Table 3**), and risk classification (**Supplementary Figure 2**). On the contrary, adding 2-h PG did not improve the model fit (traditional+2-h PG; Δ deviance: −1.31; p = 0.25; ΔC: +0.01). The addition of HbA1c marginally improved the model fit (traditional+HbA1c; Δ deviance: −4.17; p = 0.041; ΔC: +0.04). The addition of both 1-h and 2-h PG did not improve the predictive utility. Furthermore, 1-h PG improved risk classification when added to the traditional model [overall NRI 48.7 (1.2; 96.2); IDI 7.8 (1.4; 14.1)]. However, the addition of HbA1c did not improve the other metrics, and only marginal improvement was observed in IDI, whereas 2-h PG did not improve the discriminative ability appreciably in predicting hyperglycemia relapse in this patient population.

**Table 3:** Prognostic performance of 1-h PG levels for hyperglycemia relapse.

	C index	ΔC index	Net reclassification index		Integrated discrimination improvement	
			% (95% CI)	p	% (95% CI)	p
Traditional model	0.84	–	–	–	–	–
+1-h PG	0.89	0.05	48.7 [1.2; 96.2]	0.04437	7.8[1.4; 14.1]	0.01616
+2-h PG	0.85	0.01	20.2 [−28.0; 68.7]	0.41584	1.32 [−1.8; 4.4]	0.40757
+ HbA1c	0.88	0.04	35.2 [−11.9; 82.2]	0.14311	5.8 [0.3; 11.4]	0.03992
+1-h PG and 2-h PG	0.89	0.05	39.2 [−8.8; 87.2]	0.10961	7.7 [1.3; 14.1]	0.01761

The traditional model refers to age, sex, randomization group, BMI, and baseline diagnosis (HG vs. DKA) (model 4 in **Table 2**) + fasting plasma glucose.

PG, plasma glucose; DKA, diabetic ketoacidosis; BMI, body mass index.

## Discussion

This is the first study to determine the association of 1-h PG with the incidence of hyperglycemia relapse among obese Black patients presenting with DKA/SH who achieve near-normoglycemia remission. We showed that 1-PG was an independent predictor of hyperglycemia relapse in this patient population. Specifically, 1-h PG ≥199 mg/dl at the time of insulin discontinuation, even after adjustment for age, BMI, gender, presentation with DKA or SH, and HbA1c levels, was independently associated with hyperglycemia relapse. Overall, 1-h PG levels were able to predict the incidence of hyperglycemia relapse or glycemic failure better than traditionally used glucose markers such as fasting or 2-h PG levels or HbA1c.

In our analysis, 1-h PG was an independent predictor of hyperglycemia relapse even after adjusting for treatment with an oral antidiabetic agent after insulin discontinuation. Prior studies in our population of patients with DKA and SH showed that normal glucose tolerance status as defined by the ADA was not a predictor of prolonged remission (3, 7). Despite the differences in the initial presentation, the long-term clinical course between DKA and SH does not seem to differ in our previous studies (3, 6, 22). In this current study, despite adjustment for DKA and SH, a high 1-PG was predictive of hyperglycemia relapse. In our previous publication, we found that intervention with oral antidiabetic medication at the time of insulin discontinuation or near-normoglycemia remission predicted longer hyperglycemia relapse-free survival. However, this current study showed that 1-h glucose was predictive of future hyperglycemia relapse independent of having an intervention with an antidiabetic agent. Use of only fasting and 2-h glucose levels in this population may miss abnormalities detected by 1-h PG levels. Insulin secretory abnormalities are present with an abnormal 1-h PG even with normal 2-h PG levels in several different populations (23–25). A recent study showed that the rate of oral glucose absorption is one of the precipitating factors of 1-h PG excursions (26). Oral glucose absorption can be decreased by increased gastric emptying time (27), and gastric emptying time can be reduced by incretin mimetics such as glucagon-like peptide-1 receptor agonists (27, 28). While the oral glucose absorption rate has not been determined in this population, it is possible that people with the higher 1-h PG may be optimally treated with a glucagon-like peptide-1 receptor agonist (GLP-1RA).

The 1-h PG is both practical and cost-effective. The national average time for visits is 84 min (29), which lends that this testing method could potentially be implemented during a routine visit without any increase in appointment duration. As a potential method, a 75-g glucose drink could be administered by clinic staff after patient check-in, followed by the collection of the glucose level 1 h later (30). Additionally, glucose challenge testing with 1-h PG is cost-effective compared to the 2-h OGTT as a gold standard for hyperglycemia screening (31). Since there is no standard of care for optimal follow-up and treatment in our current population after insulin discontinuation, the 1-h PG could be used to determine which patients may need more intensive follow-up and earlier addition of intensification of antidiabetic therapy.

A 1-h PG >155 mg/dl has been proposed as the cutoff associated with metabolic abnormalities in several studies. However, a majority (~70%) of the participants in our study had a 1-h PG >155 mg/dl. Therefore, we performed ROC analysis to predict which 1-h PG level predicted hyperglycemia relapse with a 64% sensitivity for BG > 199 mg/day. This glucose level is potentially too high, and 1 h PG >155 mg/dl leads to metabolic abnormalities. However, 155 mg/dl was validated in patients without diabetes, and therefore, patients with diabetes may need different targets for 1-h glucose levels. Further, our definition of near-normoglycemia remission was chosen to reflect glycemic goals for people with diabetes. The definition of near-normoglycemia remission is variable depending on the study, with some studies in patients with DKA and SH using the definition of HbA1c < 6.3% and off medications for 3 months (4, 22). It is possible that a more stringent definition of near-normoglycemia remission in our study could have resulted in more participants with a 1-h PG <155 mg/dl.

The ADA published a consensus statement in 2021 defining remission as HbA1c < 6.5% and off medications for >3 months (32). We were unable to assess remission as per the ADA definition, as this study was a *post-hoc* analysis of 2 randomized controlled studies that randomized subjects to a drug or a placebo. Therefore, we used hyperglycemia relapse-free survival in this study. However, even a more rigorous HbA1c cutoff for initial remission showed that most people had dysglycemia on OGTT based on fasting and 2-h PG levels (3, 4). Further, we found that having normal glucose tolerance during OGTT does not predict time in remission while an intervention did (7). The findings from our study highlight that traditional markers used to define remission are not sufficient to predict future hyperglycemia relapse.

In conclusion, 1-h PG of ≥199 mg/dl was independently associated with hyperglycemia relapse in obese Black patients presenting with DKA/SH at the time of diagnosis of diabetes. In clinical use, adopting a 1-h PG check within 1 to 2 weeks after insulin discontinuation may offer a more effective strategy to determine which patients need an aggressive antidiabetic treatment regimen. Future studies in this population will need to be performed where the definition of remission is more stringent and includes 1-h PG and whether treatments targeted to reduce 1-h PG levels will prevent hyperglycemia relapse.

## Data Availability Statement

After reviewing the study hypothesis and detailed statistical analysis plan, the de-identified, individual participant-level data that underlie the results will be shared based on the reasonable request. All submissions should be addressed to the senior author (priyathama.vellanki@emory.edu). The applicants will be asked to sign a data access agreement before transferring the de-identified data and requested to get an IRB waiver.

## Ethics Statement

The studies involving human participants were reviewed and approved by Emory University. The patients/participants provided their written informed consent to participate in this study.

## Author Contributions

RJ conceived the study, performed the data analysis, wrote the first draft, and critically reviewed and edited it. DS critically reviewed the manuscript and made critical contributions to data analyses. DDS and GU collected data from the original two studies and critically reviewed and edited the manuscript. OO and LC critically reviewed and edited the manuscript. PV conceived the study, directed the data analysis, wrote the first draft, and critically reviewed and edited the manuscript. PV had access to all the data and is the guarantor of the work. All authors listed have made a substantial, direct, and intellectual contribution to the work and approved it for publication.

## Funding

PV is funded in part by NIH/NIDDK K23 DK 11324-01A1. This study was funded by NIH K08 DK083036 to DDS. GU is partly supported by research grants from the NIH/NATS UL1 TR002378 from the Clinical and Translational Science Award program and 1P30DK111024-01 from NIH and National Center for Research Resources.

## Conflict of Interest

DDS was Speaker Bureau and Ad hoc consultant of: Novo Nordisk and Bayer. GU has received unrestricted research support for research studies (to Emory University) from Merck, Novo Nordisk, and Dexcom Inc.

The remaining authors declare that the research was conducted in the absence of any commercial or financial relationships that could be construed as a potential conflict of interest.

## Publisher’s Note

All claims expressed in this article are solely those of the authors and do not necessarily represent those of their affiliated organizations, or those of the publisher, the editors and the reviewers. Any product that may be evaluated in this article, or claim that may be made by its manufacturer, is not guaranteed or endorsed by the publisher.

## Supplementary Material

Supplementary Figure 1 Predictive performance of five different models for hyperglycemia relapse. The different curves represent the different goodness of fit of the traditional model (age, sex, BMI, treatment allocation, and FBG) along with plasma glucose time at various time points of the oral glucose tolerance test, or HbA1c. The model containing 1-h PG showed increased discriminatory ability in predicting hyperglycemia relapse than the other biomarkers.

Supplementary Figure 2 Calibration plots of the proportion of hyperglycemia relapse within each tenth (identified using deciles) of predicted risk between traditional model and the model including 1-h PG- Hosmer-Lemeshow test.
