# Supplementary figures and images for: 1-h Glucose During Oral Glucose Tolerance Test Predicts Hyperglycemia Relapse-Free Survival in Obese Black Patients With Hyperglycemic Crises
Source: Front Endocrinol (Lausanne). 2022 Jun 2;13:871965. doi: 10.3389/fendo.2022.871965 (PMC9202609; doi:10.3389/fendo.2022.871965)

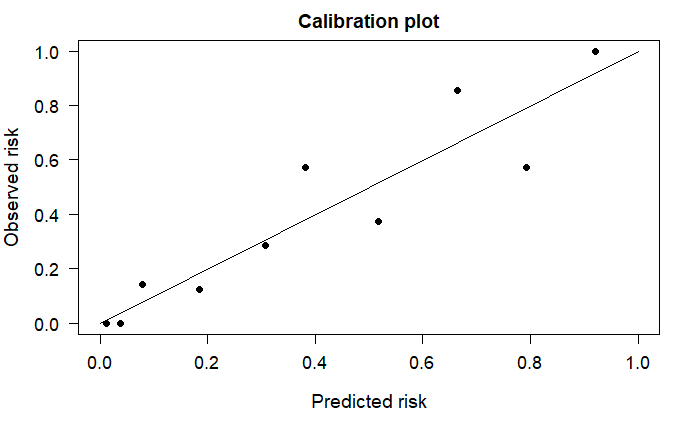

Supplement: Supplementary Figure 1 — Predictive performance of five different models for hyperglycemia relapse. The different curves represent the different goodness of fit of the traditional model (age, sex, BMI, treatment allocation, and FBG) along with plasma glucose time at various time points of the oral glucose tolerance test, or HbA1c. The model containing 1-h PG showed increased discriminatory ability in predicting hyperglycemia relapse than the other biomarkers. [file Image_1.tiff]

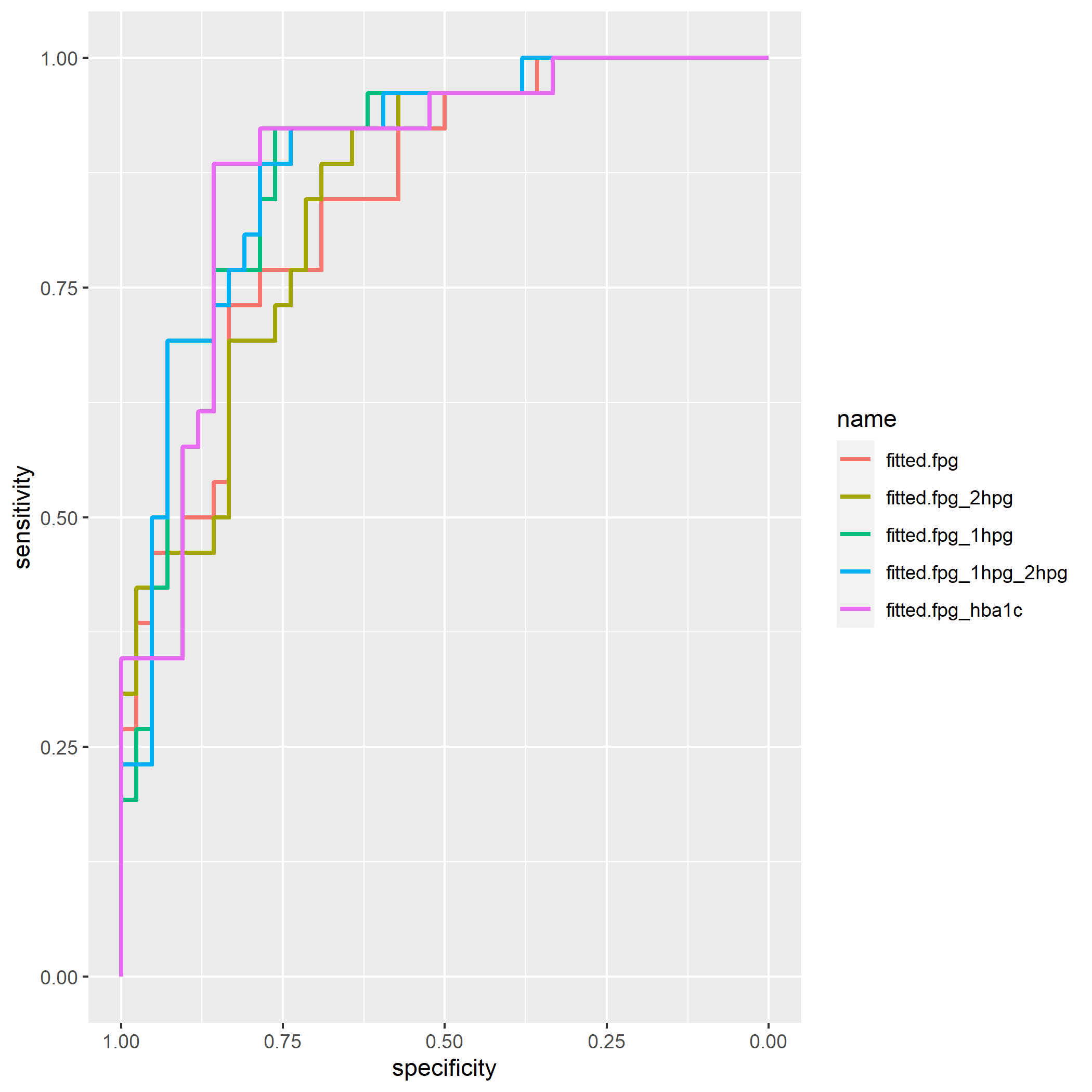

Supplement: Supplementary Figure 2 — Calibration plots of the proportion of hyperglycemia relapse within each tenth (identified using deciles) of predicted risk between traditional model and the model including 1-h PG- Hosmer-Lemeshow test. [file Image_2.tiff]
